# Supplementary material for: Greenhouse gas emissions from US irrigation pumping and implications for climate-smart irrigation policy
Source: Nat Commun. 2024 Jan 23;15:675. doi: 10.1038/s41467-024-44920-0 (PMC10803728; doi:10.1038/s41467-024-44920-0)
Supplement: Supplementary file 1 — Supplementary Information [file 41467_2024_44920_MOESM1_ESM.pdf]

Supplementary Information

Greenhouse gas emissions from US irrigation pumping and implications for climate-smart irrigation policy

Avery W. Driscoll[1*], Richard T. Conant[2], Landon T. Marston[3], Eunkyoung Choi[2], Nathaniel D. Mueller[1,2]

[1]Department of Soil and Crop Sciences, Colorado State University, Fort Collins, CO, USA

[2]Department of Ecosystem Science and Sustainability, Colorado State University, Fort Collins, CO, USA

[3]Department of Civil and Environmental Engineering, Virginia Polytechnic Institute and State University, Blacksburg, VA, USA

Contents:

Supplementary Methods
Supplementary Discussion
Supplementary Figures S1 to S7
Supplementary Tables S1 to S2
Supplementary References

Supplementary Methods

Withheld fuel expenditure data

Due to confidentiality requirements, fuel expenditure and/or area data from the Irrigation and Water Management Survey were withheld from reporting if there were a small number of irrigators in a state using a given fuel and water source. To estimate energy-use emissions for these occurrences, we calculated the difference between the total national irrigated area reported for each fuel and water source combination and the sum of the state-level values reported. The difference between the national total and the summed state-level area was assumed to be the area for which data was withheld. This approach is similar to that used by Marston et al.[1] to estimate crop production values for USDA data withheld for confidentiality. The national average expense per acre for each fuel and water source was used to estimate the total expenditures across these omitted acres. The price for each fuel was estimated by averaging prices across states for which data were withheld, after weighting the fuel price in each state by its total pumped irrigated area. The emissions factor for electricity was estimated using the same weighted averaging method across states for which data were withheld. These missing data represented a small percentage of both the total pumped irrigated area (0.48%) and the total estimated emissions (0.42%).

Spatial and temporal resolution of fuel prices from the Energy Information Agency

For diesel and gasoline, we calculated annual average on-highway retail prices for No. 2 diesel and regular gasoline, respectively, using the reported weekly data. State-level data were available in CA for diesel and in CA, TX, CO, WA, OH, MN, FL, MA, and NY for gasoline. For remaining states, prices were reported at the level of Petroleum Administration for Defense Districts (PADD), regions that contain 5 to 15 states each. Data on propane prices were collected only from October through March, and the average price across this period was used. State-level data were available for 26 states. PADD-scale data were used for the remaining states, except the US average price was used for the 5 states in the "West Coast" district, which did not have any data. Natural gas and electricity prices for industrial users were reported monthly at the state level.

Accounting for golf course irrigation

The USGS reported agricultural irrigation separately from golf course irrigation in all but 13 states: AR, LA, MO, MS, MT, ND, NE, NJ, OK, SD, TX, WI, and WY. Across states for which agriculture and golf were reported separately, water withdrawals for agricultural irrigation represented 98.5% of total withdrawals. To account for golf irrigation in the 13 states for which golf withdrawals were not separated, we multiplied water withdrawal estimates by 0.985. In West Virginia, the proportion of total irrigation water withdrawals denoted as crop irrigation was anomalously low relative to other states (0.96%, relative to the next-lowest value of 24.4% in NH and a median value of 90.2%). Therefore, we treated the irrigation water withdrawal data for WV the same way that we did for states without crop-specific irrigation data.

Missing crop-specific water application rate data

Water application rates were not available from the USDA Census data for a total of 146 state-crop combinations (accounting for 47,286 acres, or 0.1% of total irrigated area). Because water application rates are correlated among crops, we used linear regressions between water application rates for grain corn and each other crop to estimate missing rates. Six states (MA, CT, NH, VT, RI, and WV) did not have values for water application rates for grain corn. Missing data from these six states affected only 226 acres, so these observations were excluded.

Calculation of potential evapotranspiration

Daily ET_o for 2018 was calculated according to the FAO Penman-Monteith equation (Eq. S1) for a well-watered grass 0.12 m in height with fixed surface resistance of 70 seconds per meter and an albedo of 0.23[2]. Here, m is the slope of the saturation vapor pressure curve (kPa °C^{-1}), R_n is the net radiation at the crop surface (MJ m^{-2} day^{-1}); G is

the soil heat flux density (MJ m^{-2} day^{-1}), *r* is the psychrometric constant (kPa $°C^{-1}$), *U* is the wind speed (m s^{-1}) at 2 m above ground, and VPD is the vapor pressure deficit (kPa):

Eq. (S1) $$\boldsymbol{ET_o} = \frac{\mathbf{0.408} \times \boldsymbol{m} \times (\boldsymbol{R_n} - \boldsymbol{G}) + \boldsymbol{r} \times \left(\frac{\mathbf{900}}{\boldsymbol{T}+\mathbf{273}}\right) \times \boldsymbol{U} \times \boldsymbol{VPD}}{\boldsymbol{m} + \boldsymbol{r} \times (\mathbf{1}+\mathbf{0.34} \times \boldsymbol{U})}$$

Input data included daily minimum and maximum temperature from PRISM, daily minimum and maximum VPD from PRISM, downward shortwave radiation from AgERA5, wind speed at 10 m from AgERA5, and elevation from GMTED2010. R_n and *U* were derived from downward shortwave radiation and wind speed at 10 m, respectively, using the FAO conversion equations[2].

Supplementary Discussion

Discrepancies between USDA and USGS water use data

Our analysis of crop-specific emissions relies on integration of water use data from both the USDA and the USGS. Specifically, we combine county-level rates of emissions per m^3 of water withdrawn, calculated based on county-level USGS water withdrawal data, with crop-specific rates of water applied to the field, calculated based on state-level USDA data. The USDA does not have any county-level data on water use for all irrigation, and the USGS does not have any crop-specific water use data. Thus, we relied on both datasets. However, there are several methodological discrepancies between these two datasets, leading to a USDA estimate of total irrigation water use that is 35% lower than the USGS estimate. Importantly, these discrepancies only affect the crop-specific emissions estimates presented in Figure 3, and not any of the other data in the manuscript. We have made conservative adjustments to account for conveyance losses to ameliorate the discrepancy but did not force agreement between the two datasets. Thus, the crop-specific emissions estimates are likely somewhat underestimated. Specifically, the sum of our crop-specific emissions estimates is equal to 8.05 MMT CO_2e, representing 64.9% of the total national emissions estimate. For comparison, the available crop-specific data represent 74.3% of irrigated area.

Methodological differences include that the USGS data reflects water volume at the point of diversion (i.e. water withdrawn), whereas the USDA data reflects water volume at the point of application. To account for this, we adjusted the USGS surface water volumes to account for estimates of national average conveyance losses for irrigation. However, additional losses could take place between the point of withdrawal and the point of application for both surface and groundwater. Second, the USGS water use data in some states does not separate irrigation of golf courses from crop irrigation. Although we adjusted the USGS water use data to account for golf irrigation (see Supplementary Methods), we may have underestimated the amount of water used for golf course irrigation in these states. Third, the USDA data was collected in 2017, whereas the USGS data reflects water use in 2015. Fourth, the USDA methodology explicitly states that water use data are based on best estimates provided by irrigators, but that most water used for irrigation is not metered or accurately measured. Similarly, the USGS water use data are compiled by individual USGS Water Science Centers, which depend on many different local data sources and, in some cases, modeled crop water demand. Further detail on the methodology and discrepancies is available in Dickens et al. 2011[3].

Supplementary Figures

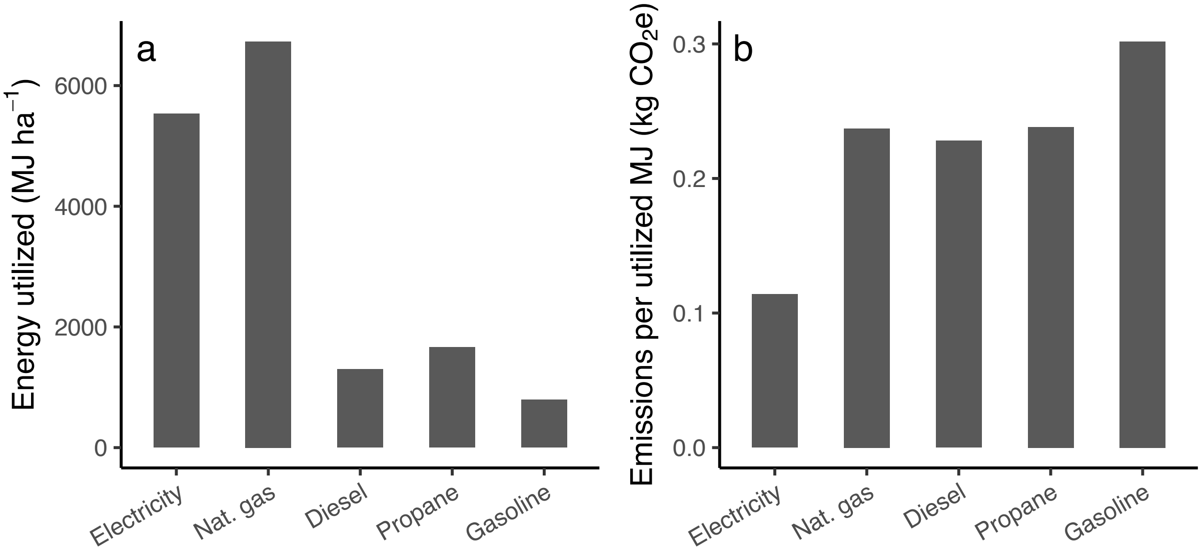

Figure S1. Average energy and emissions intensities by fuel. A) National average energy (megajoules) utilized for lift per irrigated hectare, after adjusting total pumping energy demand by fuel-specific average pump fuel efficiency estimates of 25% for propane, 23% for gasoline, 21% for natural gas, 31% for diesel, and 88% for electricity. B) Emissions factors in kilograms of CO_2e per utilized megajoule of energy, after accounting for pump fuel efficiency.

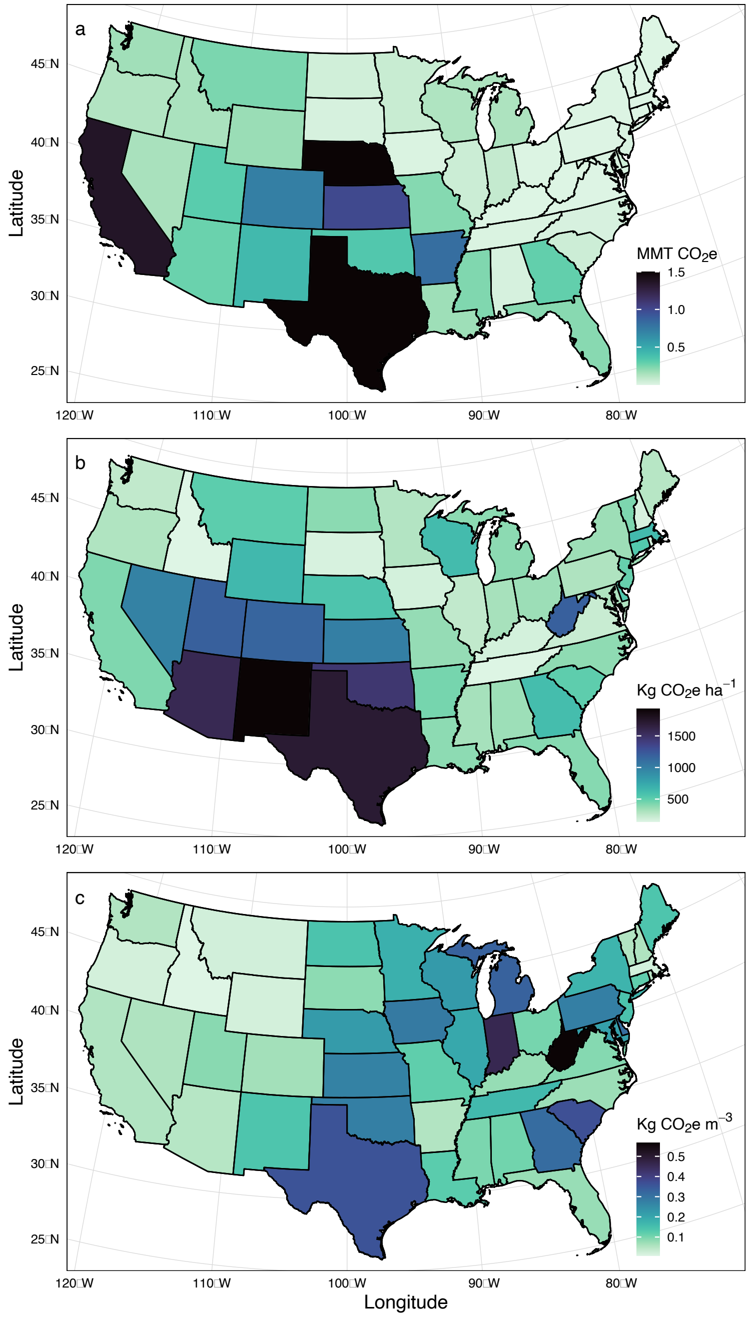

Figure S2. State-level irrigation pumping emissions and emissions intensities. A) State-level total greenhouse gas emissions (MMT CO_2e) from energy use for on-farm irrigation pumping in 2018, B) state-level greenhouse gas emissions per irrigated hectare from energy use for on-farm irrigation pumping, and C) state-level greenhouse gas emissions per cubic meter of irrigation water used. Emissions estimates for Nebraska (1.78 MMT CO_2e) and Texas (2.61 MMT CO_2e) in panel A were truncated for visibility.

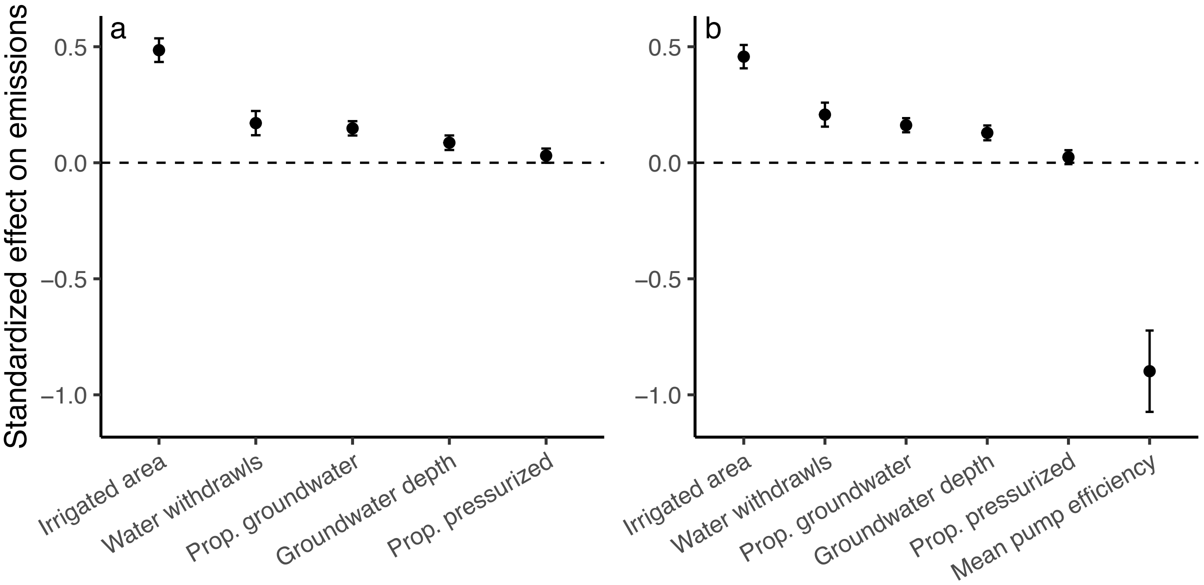

Figure S3. Drivers of irrigation pumping emissions. (A) Standardized coefficients from linear regressions of county-level greenhouse gas emissions by county-level irrigated area, water withdrawals, the proportion of irrigation water use from groundwater rather than surface water, groundwater depth, and the proportion of irrigated area utilizing pressurized irrigation systems (sprinkler or drip) rather than gravity-fed irrigation systems. For this model, $R^2 = 0.44$ and $p < 0.0001$ for all variables except the proportion of area utilizing pressurized systems, for which $p = 0.039$. In panel (B), we additionally include a state-level variable for mean pump fuel efficiency based on average pump fuel efficiency estimates of 25% for propane, 23% for gasoline, 21% for natural gas, 31% for diesel, and 88% for electricity. For this model, $R^2 = 0.46$ and $p < 0.0001$ for all variables except the proportion of area utilizing pressurized systems, for which $p = 0.074$. Coefficient estimates were qualitatively robust to the inclusion of state-level mean pump fuel efficiency. Error bars represent the 95% confidence intervals of the coefficient estimates.

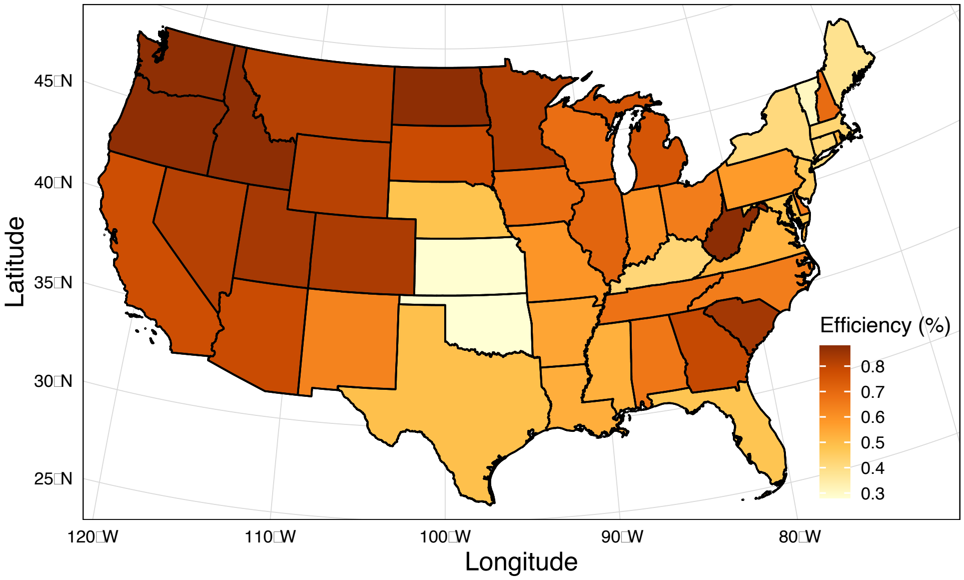

Figure S4. State-level average fuel efficiency. State-level average pump fuel efficiency, calculated as the percentage of pump energy use that is utilized for lift using average pump fuel efficiency estimates of 25% for propane, 23% for gasoline, 21% for natural gas, 31% for diesel, and 88% for electricity.

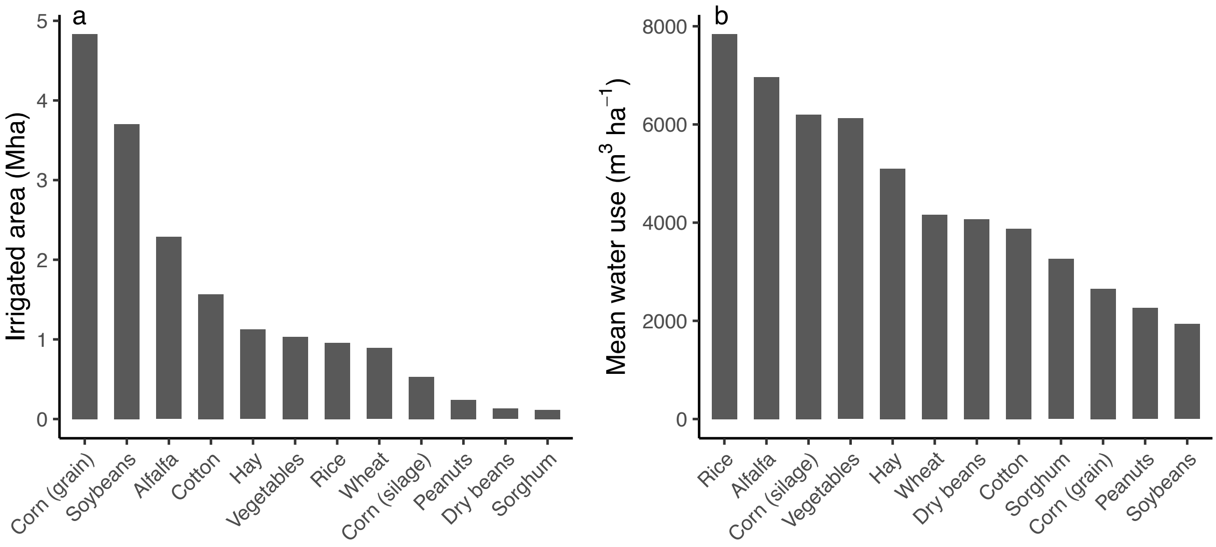

Figure S5. Crop-specific irrigated area and water use. (A) Irrigated areas in millions of hectares for each of the twelve crops that were considered in the crop-specific analysis, including only areas in counties with non-zero estimates of irrigation pumping emissions. All together, these areas account for 74.3% of total US irrigated area. (B) National average water use in cubic meters per hectare for each crop considered in the analysis.

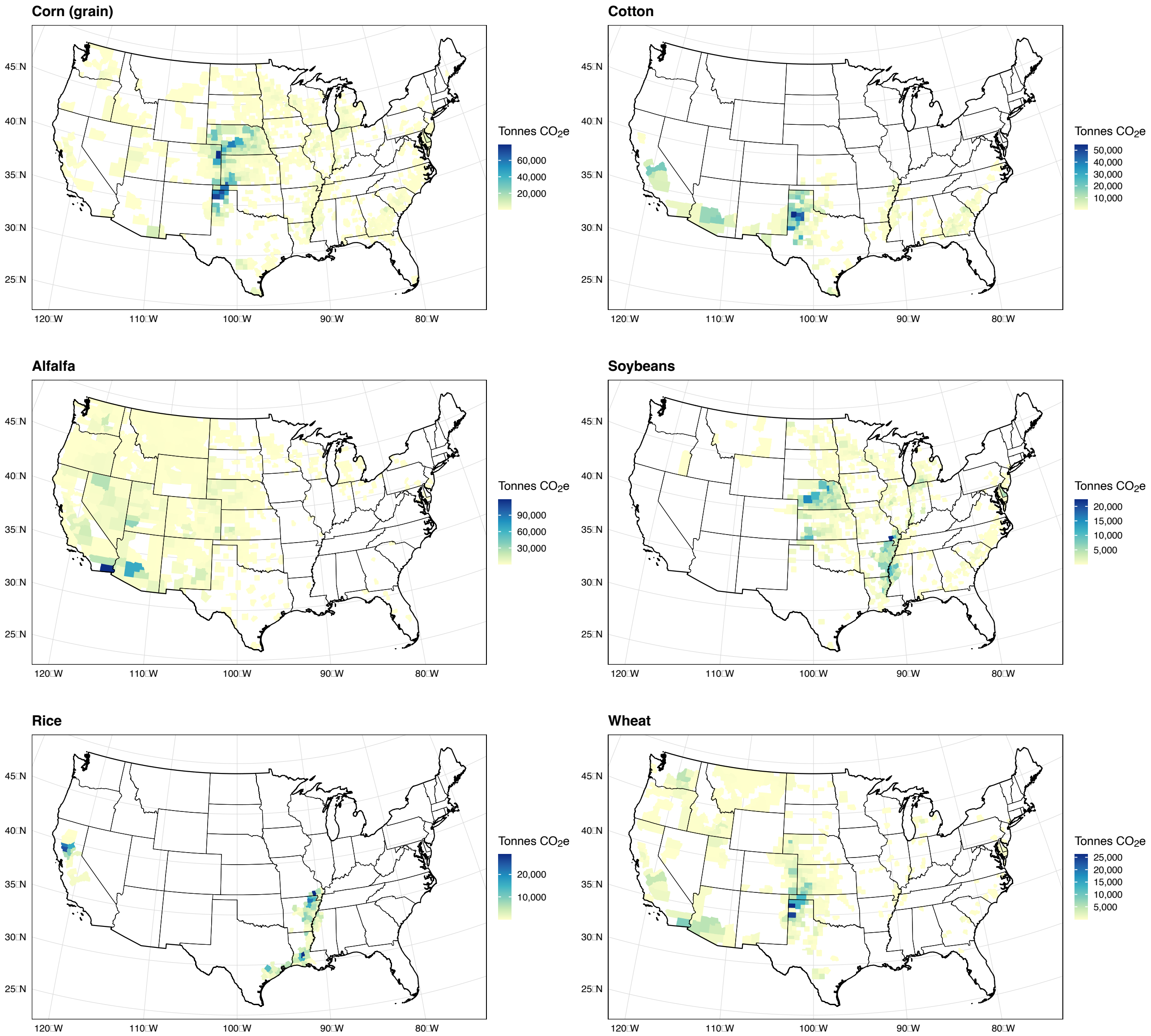

Figure S6. Crop-specific emissions maps. Greenhouse gas emissions associated with energy use for irrigation pumps for grain corn, cotton, alfalfa, soybeans, rice, and wheat. Panels are arranged in order of greatest to least total emissions and are continued in Figure S7.

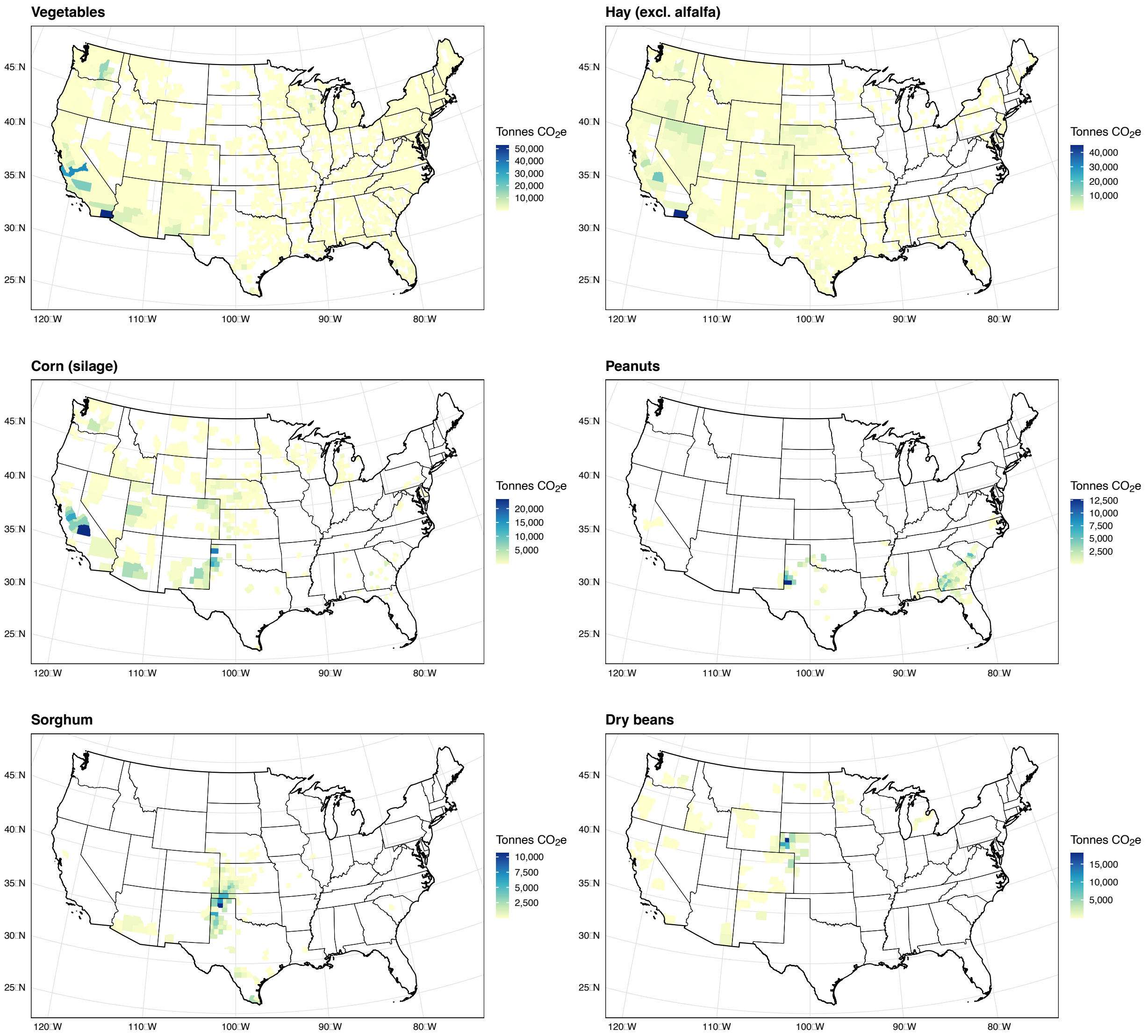

Figure S7. Crop-specific emissions maps. Greenhouse gas emissions associated with energy use for irrigation pumps for vegetables, non-alfalfa hay and haylage, corn silage, peanuts, sorghum, and dry beans. Panels are arranged in order of greatest to least total emissions.

Supplementary Tables

Table S1. Data from other studies of on-farm irrigation energy use and greenhouse gas emissions for comparison with results from the present study. For manuscripts that originally presented ranges, we report the mean value in this table. This does not reflect a comprehensive review of the literature, but rather provides a reference point for the magnitude of our results. All emissions values are in units of CO_2e. See the Supplementary References section for full citations.

**Study**	**Region**	**Scope**	**Total energy use (PJ)**	**Total emissions (MMT)**	**Energy per area (MJ/ha)**	**Energy per volume (PJ/km^3)**	**Emissions per area (t/ha)**	**Emissions per volume (MMT/km^3)**
Present	USA	Total	156	12.6	6687	1.1	0.54	0.09
		Ground-water	131	10.7	*NA*	1.7	*NA*	0.14
		Surface water	26	1.9	*NA*	0.4	*NA*	0.03
Sowby & Dicataldo[4]	USA	Total	158	*NA*	7,009	1.5	*NA*	*NA*
McCarthy et al.[5]	Kansas HPA	Ground-water	15	0.87	*NA*	3.9	*NA*	0.23
Tidwell et al.[6]	Western USA	Total*	136	*NA*	13,658	*NA*	*NA*	*NA*
Rajan et al.[7]	India	Ground-water	846	53.8	19,906	3.4	1.27	0.21
Zou et al.[8]	China	Total	*NA*	37.7	*NA*	*NA*	0.63	*NA*
Karimi et al.[9]	Iran	Ground-water	150	4.9	*NA*	3.3	*NA*	0.11
Qureshi[10]	Pakistan	Ground-water	148	3.8	43,412	3.0	1.12	0.08

*Includes electricity use only

Table S2. Publicly available data sources used for the calculation of irrigation energy use emissions.

Agency	Data set	Data used	URL
United States Department of Agriculture	2018 Irrigation and Water Management Survey: Table 13	State-scale on-farm expenditures for irrigation pump fuel	https://www.nass.usda.gov/Publications/AgCensus/2017/Online_Resources/Farm_and_Ranch_Irrigation_Survey/index.php
	2018 Irrigation and Water Management Survey: Table 32	State-scale average acre-feet of water applied per acre	
	2018 Irrigation and Water Management Survey: Table 36	State-scale average acre-feet of water applied per acre for major crops	
	2018 Irrigation and Water Management Survey: Table 14	Area utilizing solar pumps (2018)	
	2013 Farm and Ranch Irrigation Survey: Table 13	Area irrigated by fuel type (2013)	https://agcensus.library.cornell.edu/census_parts/2012-2013-farm-and-ranch-irrigation-survey/
	2013 Farm and Ranch Irrigation Survey: Table 13	Area irrigated utilizing solar pumps (2013)	
	2018 Weekly Crop Progress Reports	State-scale median planting and harvest dates for major crops	https://www.nass.usda.gov/Charts_and_Maps/Crop_Progress_&_Condition/2018/index.php
	2017 Census of Agriculture, Volume 1, Chapter 2: County Level	County-scale irrigated area (totals and by crop)	https://www.nass.usda.gov/Publications/AgCensus/2017/#full_report
	NASS Irrigation Organizations 2019 Summary	National average conveyance losses for irrigation water deliveries	https://usda.library.cornell.edu/concern/publications/x920gg177
United States Energy Information Administration	Petroleum and Other Liquids: Weekly Retail Gasoline and Diesel Prices	Weekly state or district-scale prices for retail conventional gasoline and diesel	https://www.eia.gov/dnav/pet/pet_pri_gnd_a_epmru_pte_dpgal_w.htm
	Natural Gas Prices	Weekly state-scale natural gas prices for industrial users	http://www.eia.gov/dnav/ng/ng_pri_sum_a_epg0_pin_dmcf_m.htm
	Electricity Data Browser: Average Retail Price of Electricity to Ultimate Customers by End-Use	Monthly state-scale electricity prices for industrial users	https://www.eia.gov/electricity/data/browser/#/topic/7?agg=1,0&geo=vvvvvvvvvvvvo&endsec=2&freq=M&start=200101&end=202108&ctype=linechart&ltype=pin&rtype=s&maptype=0&rse=0&pin=
	Weekly Heating Oil and Propane Prices (October – March)	Weekly state- or district-scale prices for wholesale propane	https://www.eia.gov/dnav/pet/pet_pri_wfr_a_EPLLPA_PWR_dpgal_w.htm
	Federal and State Motor Fuels Taxes	Biannual federal and state level taxes for diesel and gasoline (2018)	https://www.eia.gov/tools/faqs/faq.php?id=10&t=10#:~:text=How%20much%20tax%20do%20we,a%20gallon%20of%20diesel%20fuel%3F&text=Federal%20taxes%20include%20excises%20taxes,per%20gallon%20on%20both%20fuels.

United States Environmental Protection Agency	2021 Greenhouse Gas Emissions Factors Hub: Table 1	Emissions factors for stationary fuel combustion	https://www.epa.gov/climateleadership/ghg-emission-factors-hub
	eGRID2018 and 2020 Summary Tables: Table 3 State Output Emission Rates	State-scale emissions factors for electricity generation	https://www.epa.gov/egrid/download-data
United States Geological Society	Estimated Use of Water in the United States County-Level Data for 2015	County-scale surface and groundwater withdrawals for crop irrigation	https://www.sciencebase.gov/catalog/item/get/5af3311be4b0da30c1b245d8
National Renewable Energy Laboratory	2022 Standard Scenarios: A US Electricity Sector Outlook	Projected state-level electrical grid emissions factors	https://www.nrel.gov/docs/fy23osti/84327.pdf

Supplementary References

1. Marston, L., Ao, Y., Konar, M., Mekonnen, M. M. & Hoekstra, A. Y. High-Resolution Water Footprints of Production of the United States. *Water Resources Research* **54**, 2288–2316 (2018).
2. Allen, R. G., Pereira, L. S., Raes, D. & Smith, M. Crop evapotranspiration: guidelines for computing crop water requirements - FAO Irrigation and drainage paper 56. *Food and Agriculture Organization of the United Nations* (1998).
3. Dickens, J. M., Forbes, B. T., Cobean, D. S. & Tadayon, S. Documentation of Methods and Inventory of Irrigation Data Collected for the 2000 and 2005 U.S. Geological Survey Estimated Use of Water in the United States, Comparison of USGS-Compiled Irrigation Data to Other Sources, and Recommendations for Future Compilations: Scientific Investigations Report 2011-5166. *United States Geological Survey* (2011).
4. Sowby, R. B. & Dicataldo, E. The energy footprint of U.S. irrigation: A first estimate from open data. *Energy Nexus* **6**, 100066 (2022).
5. McCarthy, B. *et al.* Trends in Water Use, Energy Consumption, and Carbon Emissions from Irrigation: Role of Shifting Technologies and Energy Sources. *Environ. Sci. Technol.* **54**, 15329–15337 (2020).
6. Tidwell, V. C., Moreland, B. & Zemlick, K. Geographic Footprint of Electricity Use for Water Services in the Western U.S. *Environ. Sci. Technol.* **48**, 8897–8904 (2014).
7. Rajan, A., Ghosh, K. & Shah, A. Carbon footprint of India's groundwater irrigation. *Carbon Management* **11**, 265–280 (2020).
8. Zou, X. *et al.* Greenhouse gas emissions from agricultural irrigation in China. *Mitig Adapt Strateg Glob Change* **20**, 295–315 (2015).
9. Karimi, P., Qureshi, A. S., Bahramloo, R. & Molden, D. Reducing carbon emissions through improved irrigation and groundwater management: A case study from Iran. *Agricultural Water Management* **108**, 52–60 (2012).
10. Qureshi, A. S. Reducing Carbon Emissions Through Improved Irrigation Management: A Case Study from Pakistan. *Irrigation and Drainage* **63**, 132–138 (2014).
