# Supplementary material for: Greenhouse gas emissions from US irrigation pumping and implications for climate-smart irrigation policy
Source: Nat Commun. 2024 Jan 23;15:675. doi: 10.1038/s41467-024-44920-0 (PMC10803728; doi:10.1038/s41467-024-44920-0)
Supplement: Supplementary file 3 — Description of Additional Supplementary Files [file 41467_2024_44920_MOESM3_ESM.pdf]

Description of Additional Supplementary Files

File Name: Supplementary Data 1
Description: County-level greenhouse gas emissions from surface and groundwater pumping for irrigation.
